# Dosimetric advantages of a “butterfly” technique for intensity-modulated radiation therapy for young female patients with mediastinal Hodgkin’s lymphoma

**DOI:** 10.1186/1748-717X-9-94

**Published:** 2014-04-15

**Authors:** Khinh Ranh Voong, Kelli McSpadden, Chelsea C Pinnix, Ferial Shihadeh, Valerie Reed, Mohammad R Salehpour, Isidora Arzu, He Wang, David Hodgson, John Garcia, Michalis Aristophanous, Bouthaina S Dabaja

**Affiliations:** 1 Department of Radiation Oncology, Unit 97, The University of Texas MD Anderson Cancer Center, 1515 Holcombe Blvd, Houston, TX 77030, USA; 2 Department of Radiation physics, The University of Texas MD Anderson Cancer Center, Houston, TX, USA; 3 Department of Radiation Oncology, Princess Margret Hospital, Toronto, ON, Canada

**Keywords:** Hodgkin lymphoma, IMRT, Mediastinum

## Abstract

**Purpose:**

High cure rates for Hodgkin’s lymphoma must be balanced with long-term treatment-related toxicity. Here we report an intensity-modulated radiation therapy (IMRT) technique that achieves adequate target coverage for mediastinal disease while minimizing high- and low-dose exposure of critical organs.

**Methods and materials:**

Treatment plans for IMRT and conventional anteroposterior-posteroanterior (AP-PA) techniques, with comparable coverage of the planning target volume (PTV), were generated for 9 female patients with mediastinal Hodgkin’s lymphoma assuming use of inclined positioning, daily breath-hold, and CT-on-rails verification. Our “butterfly” IMRT beam arrangement involved anterior beams of 300°−30° and posterior beams of 160°−210°. Percentages of normal structures receiving 30 Gy (V_30_), 20 Gy (V_20_), and 5 Gy (V_5_) were tabulated for the right and left breasts, total lung, heart, left and right ventricles, left anterior descending coronary artery (LAD), and spinal cord. Differences in each variable, conformity index, homogeneity index, and V_107%_ between the two techniques were calculated (IMRT minus conventional).

**Results:**

Use of IMRT generally reduced the V_30_ and V_20_ to critical structures: −1.4% and +0.1% to the right breast, −1.7% and −0.9% to the left breast, −14.6% and −7.7% to the total lung, −12.2% and −10.5% to the heart, −2.4% and −14.2% to the left ventricle, −16.4% and −8.4% to the right ventricle, −7.0% and −14.2% to the LAD, and −52.2% and −13.4% to the spinal cord. Differences in V_5_ were +6.2% for right breast, +2.8% for left breast, +12.9% for total lung, −3.5% for heart, −8.2% for left ventricle, −1.5% for right ventricle, +0.1% for LAD, and −0.1% for spinal cord. Use of IMRT significantly reduced the volume of tissue receiving 107% of the dose (mean 754 cm^3^ reduction).

**Conclusions:**

This butterfly technique for IMRT avoids excess exposure of heart, breast, lung, and spinal cord to doses of 30 or 20 Gy; mildly increases V_5_ to the breasts; and decreases the V_107%_.

## Introduction

Achieving high cure rates in patients affected with Hodgkin’s lymphoma depends on balancing effective treatment with the need to minimize toxicity from chemotherapy and radiation [1-5]. Switching from MOPP (mechlorethamine, vincristine, procarbazine, and prednisone) to ABVD (doxorubicin, bleomycin, vinblastine, and dacarbazine) chemotherapy and reducing radiation field size and dose have translated into lower rates and severity of treatment-associated toxicity [4]. Radiation therapy delivered with older extended fields, namely mantle field radiation, in combination with radiation doses in excess of 40 Gy have been reported to increase the risk of developing breast cancer [6,7], especially for patients younger than 24 years [8] for whom risks approach up to 28% with long follow-up. Although radiation fields have been significantly reduced over the past two decades, using radiation to treat young female patients with mediastinal Hodgkin’s lymphoma is still challenging because of the need to target disease in the mediastinum while avoiding critical organs. We undertook this study to demonstrate that intensity-modulated radiation therapy (IMRT), delivered via a special butterfly technique beam arrangement in combination with a unique immobilization technique, can deliver the desired dose of radiation to mediastinal disease in female patients with Hodgkin’s lymphoma, with low integral doses to critical structures compared with the conventional anteroposterior and posteroanterior directed photon beam (AP-PA) technique.

## Methods

This analysis was approved by the M.D. Anderson Cancer Center institutional review board. Nine female patients with early-stage Hodgkin’s lymphoma, all with mediastinal disease without bilateral axillary disease, were identified retrospectively. All had been treated with 4–6 cycles of ABVD according to risk groups as determined at presentation. Radiation treatment simulation and planning were typically done 3–4 weeks after the final chemotherapy cycle. All patients were in complete remission at the end-of-chemotherapy as determined by positron emission tomography/computed tomography (PET/CT) restaging work-up.

### Treatment simulation

**Figure 1:**
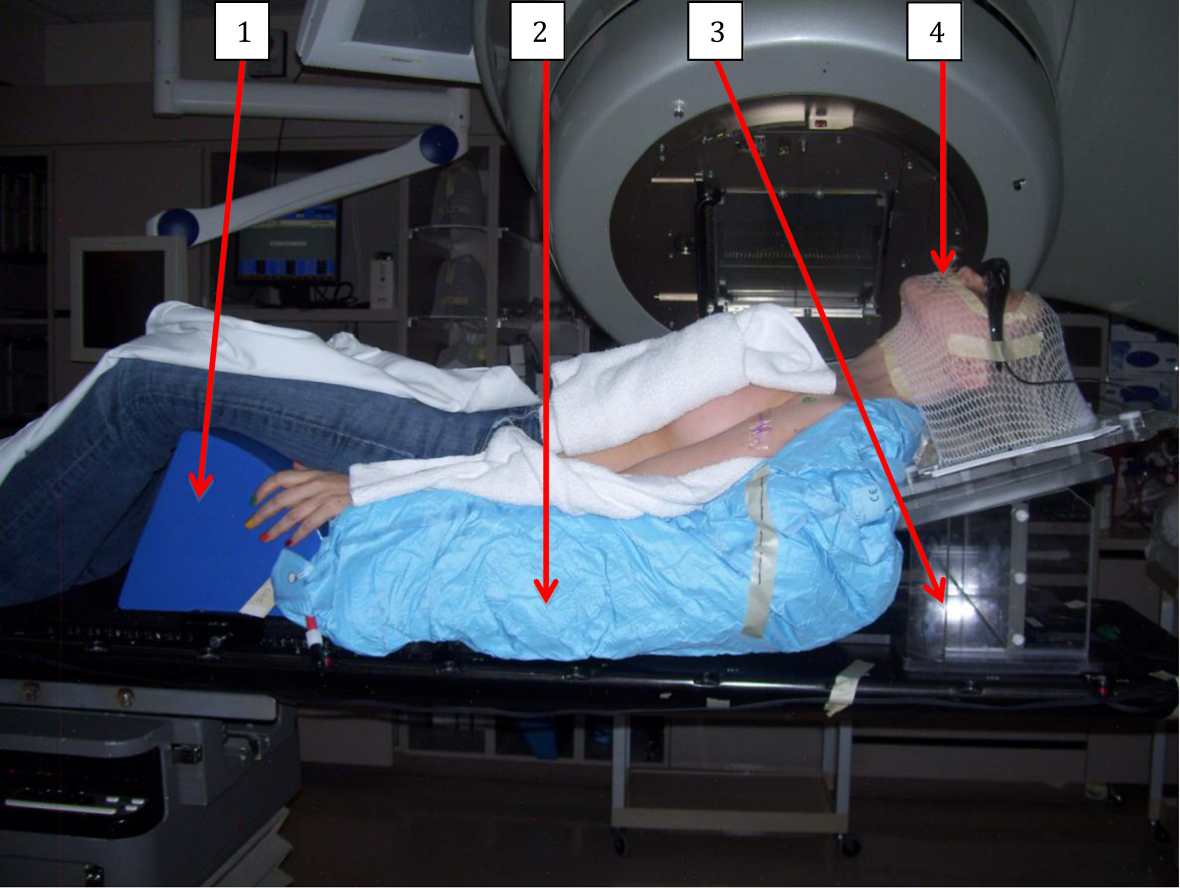
**Display of the patient’s immobilization set-up during treatment simulation.** Arrows point to hip stopper **(1)**, Vac-Lok **(2)**, inclined board **(3)**, and facemask **(4)**.

Radiation simulation and treatment were done in all cases with patients immobilized on a 10°‒15° inclined board set-up as described elsewhere [9]. Briefly, the system consisted of an inclined board on which a headrest, Aquaplast facemask (WFR/Aquaplast, Wyckoff, NJ), and a Vac-Lok bag (CIVCO Medical Solutions, Orange City, IA) are attached. The Hip-stopper (CIVCO Medical Solutions) is indexed to the table and placed at the inferior end of the bag to prevent the patient from sliding downward. Each patient was positioned on a Vac-Lok with both arms akimbo; the bag is then molded to the shape of the neck of the headrest, the spine of the inclined board, and edge of the hip stopper (Figure 1). Once the patient’s body is immobilized, an Aquaplast facemask is fashioned such that the patient’s neck is slightly extended to move the chin out of the beam entrance, especially if the neck is included in the target volume.

Because this inclined board system tends to move the patient anteriorly, the treatment isocenter is placed as posteriorly as possible to allow gantry clearance under the table for the posterior beam. In-room lasers are used to mark the coordinates of the delineated isocenter on the patient’s skin. The Real-time Position Management system (Varian Medical Systems, Palo Alto, CA) installed on a Philips computed tomography (CT) scanner, was used to monitor respiration. Before CT planning images are obtained, patients are coached on an inspiration breath-hold technique that involves the use of goggles that allow the patient to see her own tolerated breath-hold parameters. Patients are instructed to hold their breath in a comfortable deep-inspiration level to ensure reproducibility of the breath hold during daily treatments. Each patient provided at least four breath-hold gated CT scans to increase reproducibility. The breath-hold gating parameters obtained at simulation are transferred to the radiation treatment unit to guide a reproducible breath-hold with each treatment. Axial planning images were obtained in 2.5-mm intervals from the level of the orbit to below the diaphragm.

### Treatment planning

Involved-site radiation was used as defined by the 2013 National Comprehensive Cancer Network guidelines (http://www.nccn.org). Deformable image fusion of the pre-chemotherapy diagnostic contrast-enhanced CT or PET/CT with the planning CT using the angled board was performed. The attending physician contoured the clinical target volume (CTV) based initially on the pre-chemotherapy gross tumor volume, as determined by fused pre-chemotherapy diagnostic images. The CTV was expanded to account for uncertainties due to patient arm positioning during imaging and treatment planning. We generated the planning target volume (PTV) by adding a 7-mm superior/inferior expansion and a 5-mm radial expansion from the CTV (defined according to institutional guidelines based on daily measurements taken from patients treated with breath-hold and daily on-board imaging). Critical structures contoured included the total lungs, heart (from the inferior aspect of the right pulmonary artery to the apex), right ventricle, left ventricle, left anterior descending coronary artery (LAD), right breast, left breast, and spinal cord. Contours for the breast, total heart, and component cardiac structures were drawn as recommended elsewhere [10,11]. The spinal cord was contoured to include the length of the cord adjacent to the PTV with an additional 1- to 2-vertebral bodies’ length of cord as a margin.

**Figure 2:**
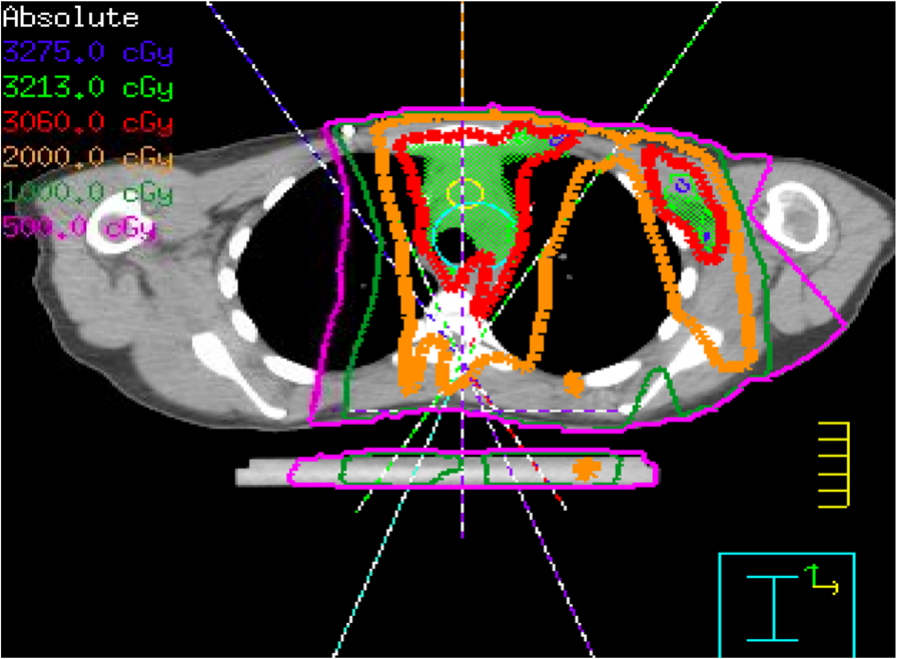
Axial CT treatment-planning scan shows beam angles for the butterfly technique.

A Pinnacle treatment planning system (version 9.0; Philips Medical Systems, Andover, MA) was used to generate radiation plans for all patients; plans targeted the PTV to a total dose of 30.6 Gy at 1.8 Gy per fraction. Both IMRT plans and conventional AP-PA conformal photon plans were created for all patients. IMRT plans were generated using a butterfly technique optimized at our institution, with 5–7 total beams restricted to anteriorly and posteriorly obliqued beam entry angles (anterior 300°‒30° and posterior 160°‒210°) (Figure 2). Beam arrangements, angles, and angle sizes were individually customized to provide tumor coverage according to each patient’s respective tumor location, while minimizing exposure to nearby critical organs. Beam angles may not have been symmetric due to well-lateralized tumor locations. Although we used individualized beam arrangements in all cases, the basic principle in the beam choice was to avoid lateral or near-lateral beams while conforming to the above-mentioned limits in the beam angles. Co-planar beams were used in all but 2 patients, for whom one non-co-planar oblique angle was necessary. One patient had right axillary disease and was treated with IMRT beam angles of 0, 20, 250, 170, and 190, directed at a second isocenter. Beams directed to this second isocenter were turned off in this study, as the purpose of this study was to evaluate plans targeting mediastinal disease. Planning objectives placed the highest priority on achieving PTV coverage, with secondary objectives being to avoid the lung, heart, and breasts. IMRT planning was generally done as follows: IMRT plans were run to obtain initial PTV coverage and critical organs doses, and then re-run with the objectives of reducing the dose to the critical organs to doses lower than those achieved with the first run, while still maintaining target coverage. Multiple planning iterations were done until an optimal plan for each patient was achieved. Conventional AP-PA plans were generated retrospectively by using 6–18 MV photons to encompass a similar percent coverage of the PTV comparable to that achieved by the IMRT plan for each individual patient. Field-in-field or multi-segmented forward planning was allowed for planning conventional AP-PA treatment. IMRT plans were further optimized for delivery by using direct machine parameter optimization. Dose-volume histograms for both techniques were generated for comparison purposes. All patients had been treated with the IMRT plans.

### Imaging verification

Daily imaging with CT-on-rails was used before radiation delivery to verify PTV coverage. Coverage of PTV by the 100% and 95% isodose lines, and hence including the CTV, were verified on the daily CT images. Shifts were made when appropriate.

### Analyses

The percentage volume (in cm^3^) of the PTV covered by 30.6 Gy was tabulated for all IMRT and comparison AP-PA plans. For each critical structure, the total baseline contoured volume was tabulated (V_PTV_). The conformity index, homogeneity index, and V_107%_ were calculated for all IMRT and comparison AP-PA plans. The conformity index was defined by the Radiation Therapy Oncology Group (RTOG) as the volume of the reference or prescription isodose divided by the target volume (CI_RTOG_ = V_RI_ /TV), which is the volume enclosed by the 100% isodose curve divided by the volume of the PTV (CI = V_100%_ /V_PTV_). CI_RTOG_ of 1, ≥1, and ≤1 correspond to an ideal conformation, a conformation with an irradiated volume greater than the PTV, and a partially irradiated PTV respectively. The homogeneity index was defined as the maximum isodose in the target divided by the reference isodose (HI_RTOG_ = Imax/RI), which is the maximum point dose divided by the prescribed dose of 30.6 Gy. HI_RTOG_ ≤ 2 is considered complaint with protocol [12]. V_107%_ was defined as the volume receiving at least 107% of the prescribed dose.

Next, the volumes that received doses of 35 Gy, 30 Gy, 25 Gy, 20 Gy, 15 Gy, 10 Gy, and 5 Gy were tabulated. The percent volume of each structure receiving the above doses was calculated by dividing the total volume receiving a specified dose by the baseline-contoured volume. Mean percent volumes receiving the above stated doses were calculated for each structure as V_35_, V_30_, V_25_, V_20_, V_15_, V_10_, and V_5_. The mean percent difference between the IMRT and AP-PA techniques was then calculated (difference = IMRT minus conventional). Wilcoxon matched-pairs signed-rank test was used to compare values between the two techniques.

## Results

**Figure 3:**
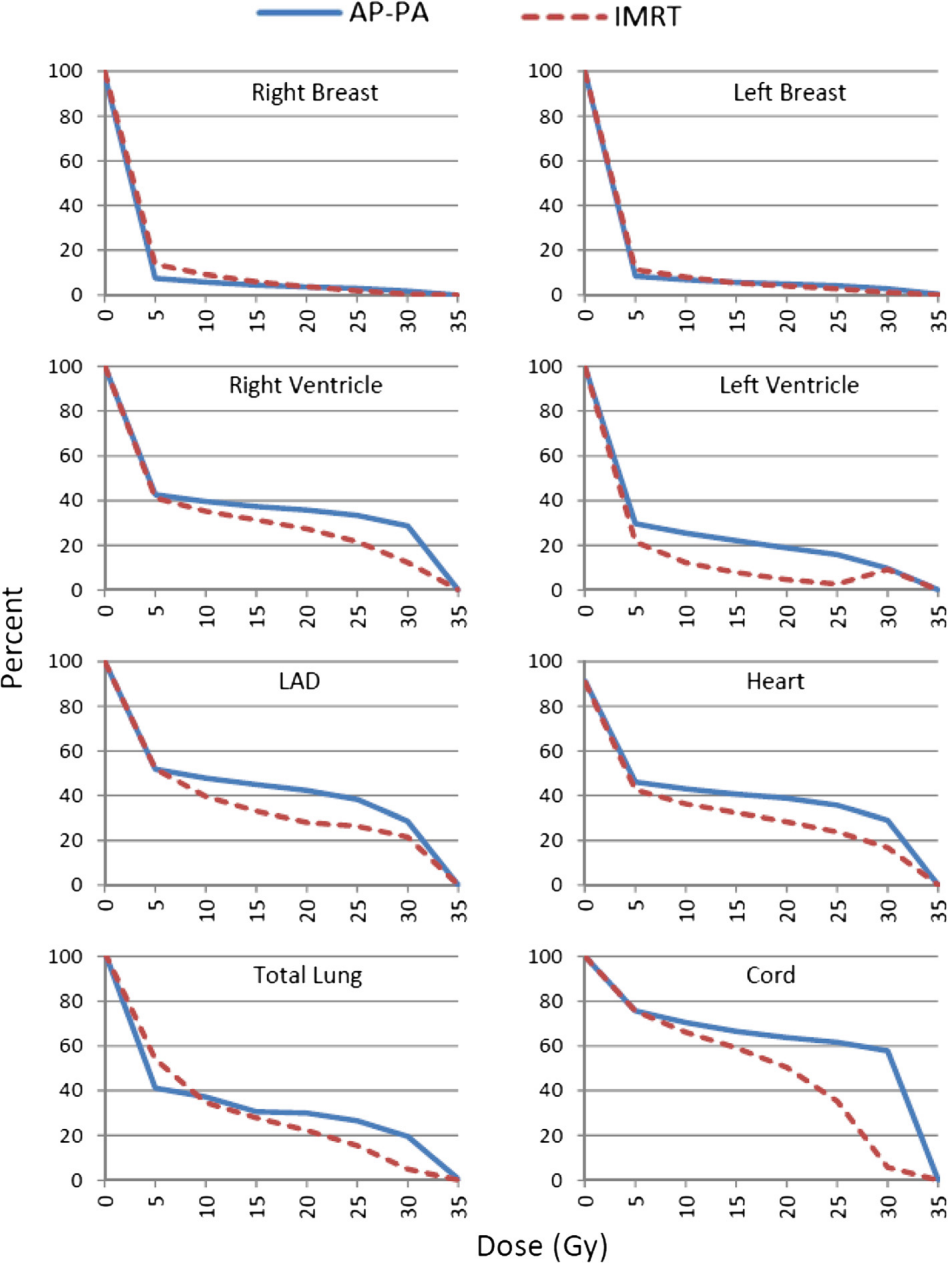
Mean volumes of organs at risk receiving 0, 5, 10, 15, 20, 25, 30, or 35 Gy.

Tables 1 and 2 list numerical results of our comparison of the two treatment planning techniques. All plans achieved acceptable target coverage as intended. IMRT plans were similar to AP-PA plans in terms of homogeneity indices (mean HI_RTOG_ = 1.15 for IMRT and 1.14 for AP-PA). IMRT plans were superior to AP-PA plans in terms of conformity indices closer to ideal value of 1 (mean CI_RTOG_ = 1.10 for IMRT and 1.66 for AP-PA plans) and smaller V_107%_ (753.8 cm^3^ reduction). Figure 3 graphically depicts the results comparing mean percent volumes of normal structures receiving radiation doses of 0 Gy to 35 Gy in 5-Gy increments for both techniques.

**Table 1:** Mean percent volumes of organs at risk receiving 30, 25, 20, or 5 Gy and mean dose to organs at risk via anteroposterior-posteroanterior (AP-PA) or butterfly intensity-modulated radiation therapy (IMRT) techniques

**Organ at risk**		**IMRT**	**AP-PA**	**Difference**	P value*
Right breast	V_30_	0.5%	1.9%	−1.4%	0.01
	V_25_	1.9%	3.1%	−1.2%	0.03
	V_20_	3.8%	3.7%	0.1%	0.44
	V_5_	13.7%	7.5%	6.2%	0.01
	Mean Dose, Gy	2.28	1.92	0.36	0.26
Left breast	V_30_	1.2%	2.9%	−1.7%	0.02
	V_25_	2.8%	4.2%	−1.4%	0.01
	V_20_	4.0%	4.9%	−0.9%	0.01
	V_5_	11.2%	8.4%	2.8%	0.01
	Mean Dose, Gy	2.42	2.38	0.04	0.41
Right ventricle	V_30_	12.3%	28.7%	−16.4%	0.01
	V_25_	21.7%	33.4%	−11.7%	0.01
	V_20_	27.4%	35.8%	−8.4%	0.01
	V_5_	41.2%	42.7%	−1.5%	0.17
	Mean Dose, Gy	10.25	12.16	−1.91	0.09
Left ventricle	V_30_	7.3%	9.7%	−2.4%	0.28
	V_25_	2.6%	15.8%	−13.2%	0.01
	V_20_	4.6%	18.8%	−14.2%	0.01
	V_5_	21.5%	29.7%	−8.2%	0.21
	Mean Dose, Gy	4.13	7.72	−3.59	0.02
Left anterior descending artery	V_30_	21.5%	28.5%	−7.0%	0.28
	V_25_	26.4%	38.2%	−11.8%	0.01
	V_20_	28%	42.2%	−14.2%	0.01
	V_5_	51.8%	51.7%	0.1%	0.55
	Mean Dose, Gy	10.44	14.46	−4.02	0.02
Heart	V_30_	16.8%	29%	−12.2%	0.01
	V_25_	23.8%	35.7%	−11.9%	0.01
	V_20_	28.3%	38.8%	−10.5%	0.01
	V_5_	42.5%	46.0%	−3.5%	0.34
	Mean Dose, Gy	11.52	14.26	−2.74	0.01
Total lung	V_30_	4.8%	19.5%	−14.6%	0.01
	V_25_	15.4%	26.5%	−11.1%	0.01
	V_20_	22.2%	29.9%	−7.7%	0.01
	V_5_	54.0%	41.1%	12.9%	0.17
	Mean Dose, Gy	9.25	11.31	−2.06	0.01
Spinal cord	V_30_	5.6%	57.8%	−52.2%	0.01
	V_25_	35.3%	61.7%	−25.4%	0.01
	V_20_	50.4%	63.8%	−13.4%	0.01
	V_5_	75.6%	75.7%	−0.1%	0.86
	Mean Dose, Gy	17.16	21.93	−4.77	0.01

*P values generated by the Wilcoxon match-pairs signed-rank test for non-parametric comparisons.

**Table 2:** **Comparison of the mean conformity index, homogeneity index, V**_**107%**_**, PTV dose, and monitor units delivered per fraction for anteroposterior-posteroanterior (AP-PA) or butterfly intensity-modulated radiation therapy (IMRT) techniques**

	**IMRT**	**AP-PA**	**Difference**	P value*
RTOG Conformity Index (V_100%_/V_PTV_)	1.10	1.66	−0.56	0.37
RTOG Homogeneity Index (Imax/RI)	1.15	1.14	0.01	0.89
V_107%,_ cm^3^	47.7	801.5	−753.8	0.01
PTV dose, Gy	31.39	32.02	−0.63	0.01
Monitor Units per Fraction	797	208	589	0.01

Imax is the maximum isodose or maximum point dose. RI is the reference isodose or 30.6 Gy.

*P values generated by the Wilcoxon match-pairs signed-rank test for non-parametric comparisons.

**Figure 4:**
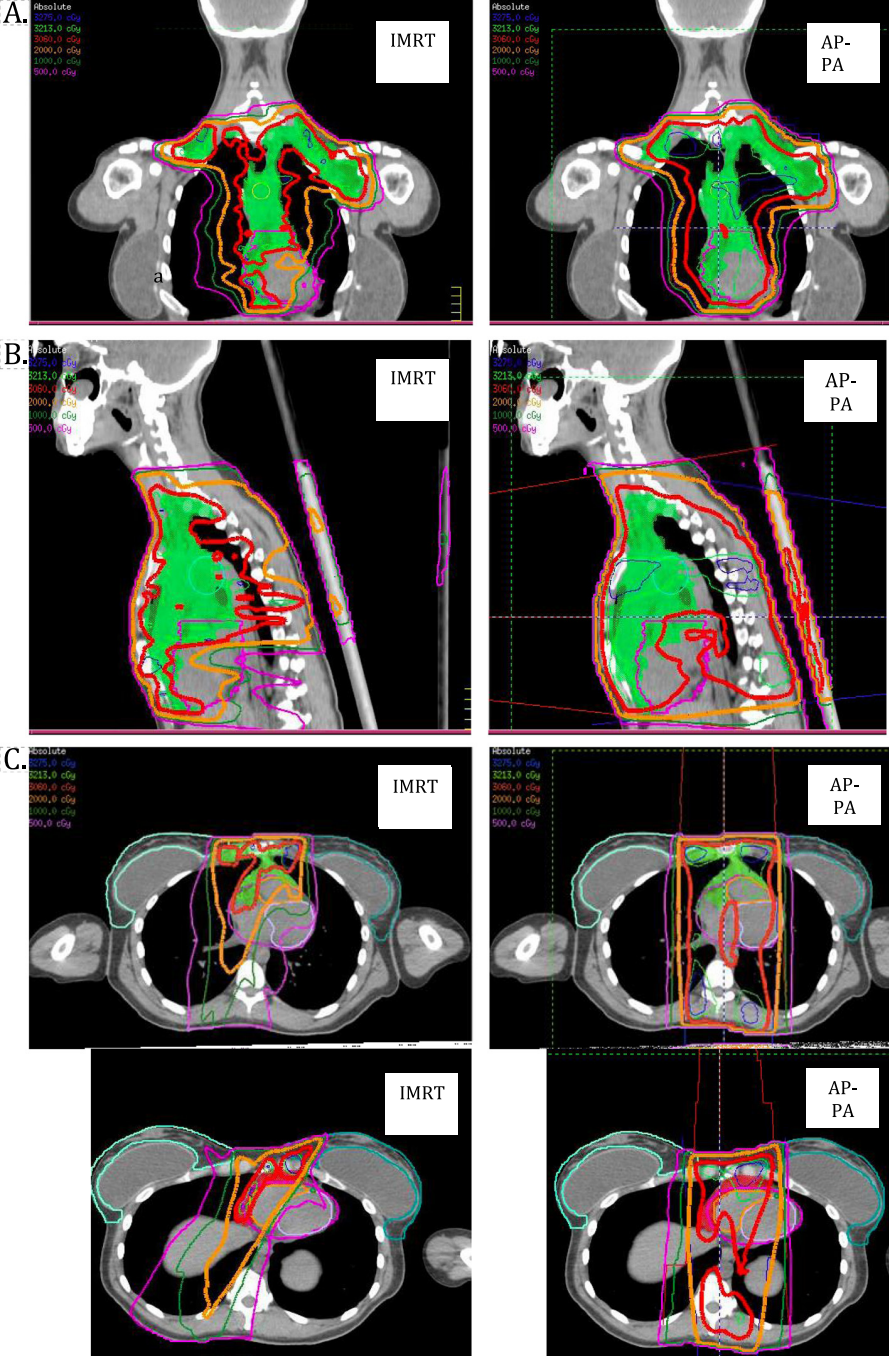
**Coronal (A), sagittal (B), and axial (C) views of a butterfly intensity-modulated radiation therapy (IMRT) plan (left) and plan using anteroposterior-posteroanterior (AP-PA) photon beams (right).** Red isodose lines represent 30.6 Gy; orange, 20 Gy; green, 10 Gy; and purple 5 Gy. The clinical target volume (shaded green) includes initial sites of nodal involvement. The Butterfly IMRT plan limits the 30.6-Gy dose to the breasts (panel **C**).

Mean V_30_, V_20_, and V_5_ values for the right and left breasts were quite similar with either technique, with corresponding [IMRT minus AP-PA] differences of −1.4%, +0.1, and +6.2%; corresponding differences for the left breast were −1.7%, −0.9%, and +2.8%. IMRT led to more substantial reductions in V_30_, V_20_, and V_5_ for the entire heart and its component structures than was true for the breasts; the corresponding differences between techniques were −12.2%, −10.5%, and −3.5% for the entire heart; −2.4%, −14.2%, and −8.2% for the left ventricle; −16.4%, −8.4%, and −1.5% for the right ventricle; −7%, −14.2%, and +0.1% for the LAD. IMRT also led to lower lung V_30_ and V_20_ but higher V_5_ values than did AP-PA, with differences of −14.6%, −7.7, and +12.9%. Finally, IMRT produced substantial reductions in spinal cord V_30_ and somewhat lesser reductions in spinal cord V_20_ and V_5_, with the differences being −52.2%, −13.4%, and −0.1%. Comparison of corresponding IMRT minus AP-PA mean organ doses supports that doses to right and left breasts were quite similar with either technique (+0.36 Gy to the right breast, +0.04 Gy to the left breast). However, IMRT led to more substantial reductions in overall mean dose to the heart and its components (−2.74 Gy heart; −3.59 Gy left ventricle, −1.91 Gy right ventricle, − 4.02 Gy LAD), total lung (−2.06 Gy), and spinal cord (−4.77 Gy) (Figure 4).

Finally, the AP-PA technique led to consistently higher V_107%_, higher mean PTV dose, and worse conformity indices, indicating more “hot spots” and an irradiated volume greater than the PTV, than from the IMRT plans (Table 2).

## Discussion

We demonstrated here that using IMRT, with a special beam arrangement, to deliver consolidative radiation therapy for mediastinal disease in female patients with Hodgkin lymphoma could effectively reduce the exposure of normal structures to high radiation doses compared with the conventional AP-PA technique. This reduction was associated with acceptable increases in low integral dose exposure to the breasts, heart, and lungs. The IMRT plans had conformity indices closer to 1 (mean CI = 1.1) and lower V_107%_ (mean −753.8 cm^3^) values, both of which are highly desirable. The benefit derived from IMRT arises from its ability to “paint” doses in such a way as to localize high doses over target structures while avoiding normal tissues by using multi-leaf fields. One of the main disadvantages of IMRT, the low integral doses delivered to critical structures (“low-dose bath”), has led to underuse of this technique for cases such as those described here. However, we demonstrated that using (1) individually optimized beam arrangements (as opposed to automatic and equally distanced beams) for (2) optimally immobilized patients (with the breast and heart displaced out of the immediate radiation field by using an angled board and breath-hold technique), and (3) consistent application of planning objectives, we could minimize low integral dose to normal structures such as the breast in young female patients. This minimization is particularly important in view of the risk of breast cancer associated with radiation doses as low as 5 Gy. Modern radiation therapy techniques have also been shown to reduce the risk of breast cancer [6,13] as a direct result of reducing both the radiation field size (through elimination of extended radiation fields) and the radiation doses (from 50 Gy to 30–20 Gy) [14].

Nevertheless, some radiation oncologists are reluctant to use IMRT for female patients with Hodgkin lymphoma because of concerns over providing high-dose exposure to normal structures and excessive low-dose radiation dose exposure to large volumes of normal tissues such as the breast. Although the risk of breast cancer is clearly dose-dependent, the relationship between low-dose radiation and carcinogenesis is more speculative [1,15][16,17]. Weber et al. [18], in using a nonlinear dose-risk model to compare IMRT with conventional techniques, concluded that IMRT may increase the estimated risk of radiation-induced cancers compared with other conformal techniques. Certainly traditional IMRT beam arrangements involving 9 fixed and equally distributed beams from the starting gantry position of 0° produce a low-dose bath to the all surrounding critical organs; however, this is exactly what we tried to avoid in our patients. In our study, the mean volume of both breasts receiving a low dose (5 Gy) was slightly higher from IMRT than from a conventional technique (6.2% for the right breast and 2.8% for the left), which we contend can be justified by the reductions in high-dose exposure (e.g., V_30_ values of −1.4% to 1.7% for the breasts, −12% for heart, −15% for lung, and −52% for spinal cord).

Another advantage of IMRT that cannot be achieved with AP-PA is the ability to “tailor” and conform the dose in difficult or complex anatomic locations. For example, the common involvement of mediastinal nodes on the right side of the heart (Figure 4) is challenging for conventional radiation planning, because AP-PA beams unavoidably enter through the breasts, whereas IMRT offers the flexibility of changing the beam angle to avoid the breast (Figure 4, panel C). In this patient case, her disease was well lateralized to the right of heart and anteriorly. In order to spare the left ventricle, a beam was angled to the right to provide target coverage, and spare the left ventricle as well as right breast, at the expense of some dose to the left breast. Notably, IMRT may not be the best technique for every patient; for example, when the target volume includes the bilateral axillae as well as the mediastinum, it is difficult to achieve acceptably low lung doses with IMRT, and planning with conventional AP-PA techniques is better in such cases.

Fiandra et al., with a similar concept to ours compared IMRT with three-dimensional conformal techniques for Hodgkin lymphoma and showed that IMRT produced similarly superior target coverage and organs-at-risk sparing [19]. However, that study treated smaller volumes with involved-node radiation, did not displace the breast out of the radiation field with supine immobilization, nor use breath-hold techniques to reduce internal organ motion.

Our conclusions from this analysis are as follows: IMRT is an inverse planning process that is the end result of creating targets, creating avoidance structures, and setting planning objectives; nevertheless, critical analysis and modification of this process are necessary to create the desired target coverage, as we did here. After having used IMRT for more than a decade, we have come to realize that the radiation planning process must be tailored carefully in each individual situation, especially those involving tumors in complex anatomic locations. Radiation planning should not be automated, and beam angles should be carefully selected on an individual basis to best suit each patient’s planning. Finally, we conclude that use of “butterfly” IMRT beam arrangements in radiation planning, in conjunction with immobilization with an inclined board and breath-hold during radiation treatment, can (1) adequately cover the target volume, (2) reduce high doses to nearby critical organs (such as the bilateral breasts in female patients), and (3) keep the low integral dose (e.g., 5 Gy) at acceptable levels in comparison with conventional AP-PA techniques.

## Consent

Consent was obtained from patient(s) for the publication of this report and representative treatment planning images and plans included in this manuscript.

## Competing interests

The authors declare that they have no competing intersts.

## Authors’ contributions

KRV contributed to the data acquisition, statistical testing, data analysis, and manuscript drafting. KM and JG contributed to data acquisition. KM, CPC, VR, and MS contributed to data analysis and drafting of the manuscript. DH, IA, HW, MA, and FS contributed to drafting of the manuscript. BSD formulated the study conception, design, and contributed to data analysis as well as drafting of the manuscript. All authors provided approval of the final manuscript.

## Research support

Cancer Center Support (core) Grant CA016672 to The University of Texas MD Anderson Cancer Center.

Summary: This work shows how a special IMRT beam arrangement when compared to conventional radiation can achieve the desired dose to the target while keeping an acceptable low dose to the critical structures.
